# Conflict of mitochondrial phylogeny and morphology-based classification in a pair of freshwater gastropods (Caenogastropoda, Truncatelloidea, Tateidae) from New Caledonia

**DOI:** 10.3897/zookeys.603.9144

**Published:** 2016-07-06

**Authors:** Marlen Becker, Susan Zielske, Martin Haase

**Affiliations:** 1 Vogelwarte, Zoologisches Institut und Museum, Ernst-Moritz-Arndt-Universität Greifswald, Soldmannstraße 23, D-17489 Greifswald, Germany

**Keywords:** Geometric morphometrics, hybridization, incomplete lineage sorting, introgression, morphology, shape, South Pacific, taxonomy

## Abstract

Morphological classification and mitochondrial phylogeny of a pair of morphologically defined species of New Caledonian freshwater gastropods, *Hemistomia cockerelli* and *Hemistomia fabrorum*, were incongruent. We asked whether these two nominal species can be unambiguously distinguished based on shell morphology or whether the taxonomic discrepancy inferred from these character types was reflected in the variation of shell morphology. Our investigations were based on phylogenetic analyses of a fragment of the mitochondrial cytochrome c oxidase subunit I, geometric morphometric analyses as well as micro computer tomography. The species presorted to morphospecies by eye overlapped in shell shape. However, statistically, all shells were correctly assigned, but not all of them significantly. Qualitatively, both nominal species can be unambiguously distinguished by the presence/absence of a prominent denticle within the shell. In the phylogenetic analyses, individuals from three populations clustered with the “wrong” morphospecies. In the absence of data from multiple loci, it was assumed for the single specimen from one of these populations that its misplacement was due to a recent hybridization event, based on its very shallow position in the tree. For the other two cases of misplacement neither introgression nor incomplete lineage sorting could be ruled out. Further investigations have to show whether the morphological overlap has a genetic basis or is due to phenotypic plasticity. In conclusion, despite their partly unresolved relationships *Hemistomia cockerelli* and *Hemistomia fabrorum* may be considered sister species, which are reliably diagnosable by the presence or absence of the denticle, but have not yet fully differentiated in all character complexes investigated.

## Introduction

Conflict in phylogenetic signal between different characters, e.g. between different genes, between mitochondrial (mt) and nuclear (nc) DNA, or between genes and morphology, is commonly observed across a wide range of taxa (e.g., Wiens and Hollingsworth 2000; Shaw 2002; Pelser et al. 2010; Sauer and Hausdorf 2010; Debiasse et al. 2014; Sharma et al. 2015). This conflict may be due to a number of reasons including selection, convergent evolution, various forms of reticulate evolution, cryptic species, demography, inhomogeneous evolutionary rates, incomplete lineage sorting, and unresolved taxonomy (Felsenstein 1978; Maddison 1997; Funk and Omland 2003; Seehausen 2004; Arnold 2006; Mallet 2007; Nosil 2012). In a recent phylogenetic analysis of tateid gastropods from New Caledonia, Zielske and Haase (2015) discovered incongruent topologies of trees based on mitochondrial (COI, 16S rRNA) and nuclear (ITS2) gene sequences regarding a pair of nominal species, *Hemistomia cockerelli* (Haase and Bouchet 1998) and *Hemistomia fabrorum* (Haase and Bouchet 1998). Not only were the sets of sequence data in conflict, also classification based on shell morphology did not match the DNA data. Zielske and Haase (2015) assumed that introgression through hybridization may be responsible for these conflicts, however, postponed a more definite statement to a more comprehensive analysis involving more populations and more specimens per locality.

**Figure 1.**
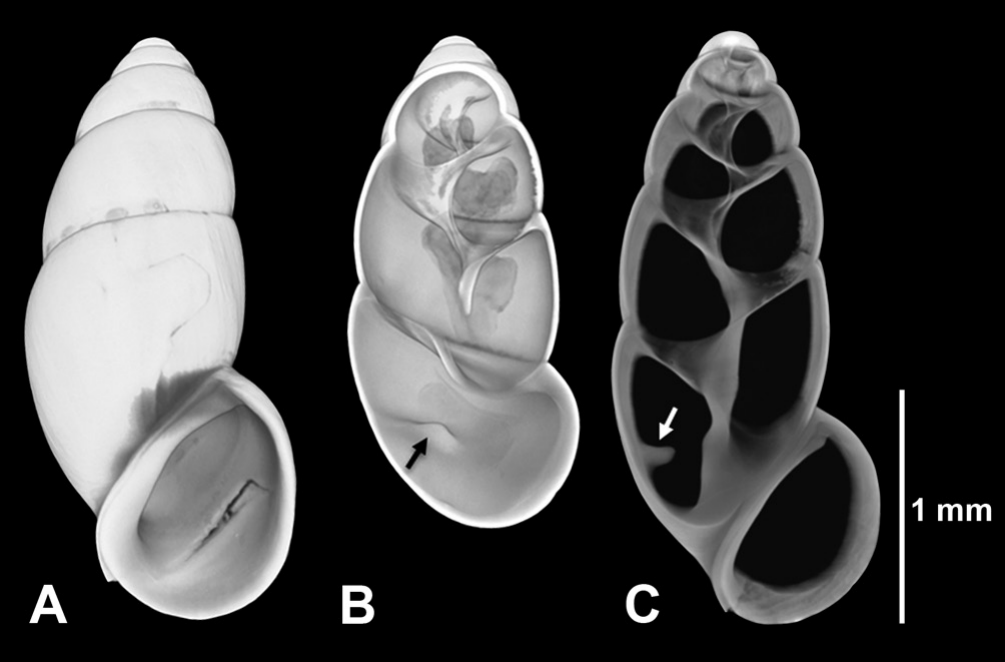
*Hemistomia cockerelli*, paratype. **A** Whole shell slightly tilted for better recognition of denticle exposed after digitally opening in **B** (arrow) **C** Longitudinal section in upright position showing denticle (arrow).

**Figure 2.**
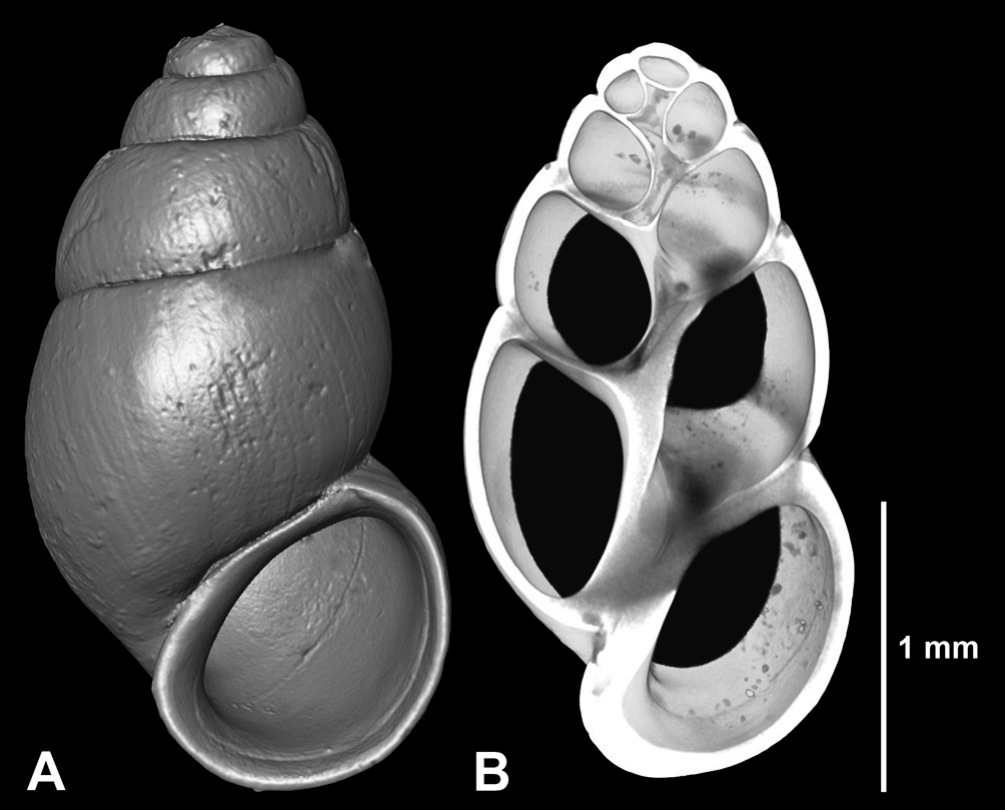
*Hemistomia fabrorum*, topotype. **A** Whole shell **B** Longitudinal section.

These investigations were initially the goal of the present account. Unfortunately, we could not consistently amplify ITS2 across the entire, enlarged data set. Therefore, we had to restrict this analysis to a comparison of COI-phylogeny and shell morphology. Typical *Hemistomia cockerelli* have a slender-conical shell whereas *Hemistomia fabrorum* is much broader. In addition, *Hemistomia cockerelli* is characterized by a prominent palatal denticle c. 1/3 whorl behind the outer lip (Figs 1, 2). Anatomically, these two species are very similar (Haase and Bouchet 1998). However, the variation within and among populations of each taxon is considerable and identification based on shell shape alone may be ambiguous. Both species occur in springs as well as small streams and have fairly broad, overlapping ranges. Occasionally, they are encountered in sympatry (Haase and Bouchet 1998, present paper: population 38). Hence, the question guiding our present analysis was whether these two nominal species can be unambiguously distinguished from each other based on shell morphology. In other words, we asked whether the conflict between shell-based classification and DNA-based phylogenies (Zielske and Haase 2015) is reflected in shell morphology and how this conflict may be biologically explained and interpreted taxonomically.

## Material and methods

### Material

**Figure 3.**
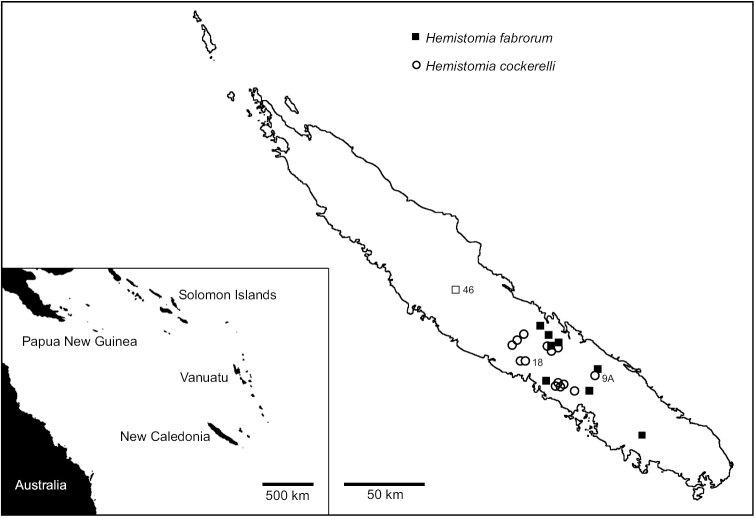
Map of New Caledonia showing sampling localities. Samples “misplaced” in phylogenetic analyses identified by population numbers.

Most specimens examined in this study were collected in 2012 at 22 localities in New Caledonia (Table 1, Fig. 3; Zielske and Haase 2015). Presorting of the collected animals to morphospecies was made by eye and in case of population 46 deviated from our previous paper. 37 paratypes (Haase and Bouchet 1998) of *Hemistomia cockerelli* included in morphometric analyses were borrowed from the Museum National d’Histoire Naturelle Paris (MNHN-IM-2012-2694). Altogether 324 individuals from 24 sites were used for morphometric analyses. 108 thereof from 20 sites were used for phylogenetic analyses. The COI-sequence of a specimen from population 18 was taken from Zielske and Haase (2015). Sequences were submitted to GenBank and received accession numbers KT203603 - KT203710. Specimens included in the recent study are encoded as follows: “Population.Specimen Number”, for instance “1A.01” represents specimen number one from population 1A.

**Table 1.** Material investigated. N_gen_, number of genetically investigated specimens; N_mor_, number of morphologically investigated specimens; Pop#, population number; Pt, paratypes.

Pop#	Species	Latitude Longitude	N_mor_	N_gen_
1A	*Hemistomia fabrorum*	22°08'59.0"S, 166°29'10.6"E	25	10
6B	*Hemistomia fabrorum*	21°48'08.0"S, 166°04'14.6"E	36	9
8	*Hemistomia fabrorum*	21°44'32.1"S, 166°05'20,6"E	4	0
9A	*Hemistomia cockerelli*	21°44'30.9"S, 166°05'57.9"E	3	2
10	*Hemistomia fabrorum*	21°42'55.4"S, 166°07'21.1"E	15	8
11	*Hemistomia cockerelli*	21°48'16.8"S, 166°00'00.8"E	26	7
13	*Hemistomia cockerelli*	21°47'30.8"S, 165°54'31.6"E	20	7
14	*Hemistomia cockerelli*	21°47'30.8"S, 165°54'38.7"E	11	8
15A	*Hemistomia cockerelli*	21°47'24.4"S, 165°54'51.2"E	6	1
16	*Hemistomia cockerelli*	21°47'24.4"S, 165°54'51.2"E	10	2
17	*Hemistomia cockerelli*	21°39'52.8"S, 165°43'10.3"E	11	2
18	*Hemistomia cockerelli*	21°39'40.6"S, 165°43'06.9"E	22	1
25B	*Hemistomia fabrorum*	21°34'15.7"S, 165°49'41.2"E	11	9
28	*Hemistomia fabrorum*	21°31'07.4"S, 165°48'20.0"E	11	10
30C	*Hemistomia cockerelli*	21°34'21.6"S, 165°41'02.5"E	9	1
31	*Hemistomia cockerelli*	21°33'33.5"S, 165°42'11.3"E	7	2
32	*Hemistomia cockerelli*	21°34'55.9"S, 165°40'16.7"E	6	0
36	*Hemistomia cockerelli*	21°38'22.1"S, 165°51'37.5"E	9	3
38B	*Hemistomia fabrorum*	21°38'09.3"S, 165°51'52.7"E	11	9
38C	*Hemistomia cockerelli*	21°38'09.3"S, 165°51'52.7"E	10	6
39	*Hemistomia fabrorum*	21°37'56.1"S, 165°51'54.4"E	6	2
41	*Hemistomia cockerelli*	21°38'12.3"S, 165°51'34.1"E	5	0
46	*Hemistomia fabrorum*	21°14'30.2"S, 165°16'30.8"E	13	9
Pt	*Hemistomia cockerelli*	21°49.2’S, 166°56.6’E	37	0

### Shell morphology

All 324 individuals were investigated for the presence/absence of a denticle 1/3 whorl behind the outer lip, which has been described as diagnostic for *Hemistomia cockerelli* (Haase and Bouchet 1998), under a dissecting microscope. For documentation, four specimens were selected for micro-computed X-ray tomography (μCT) - one paratype of *Hemistomia cockerelli*, one topotype of *Hemistomia fabrorum*, and one specimen each from morphologically intermediate populations 9A and 46 (see below). Scans were performed using an XRadia Micro XCT-200 (Carl Zeiss X-ray Microscopy Inc., Pleasanton, USA). The samples were placed in pipette tips glued on an insect pin. Each shell was scanned for 1 h at 40 kV and 8 W at four times magnification. Image stacks were processed and three dimensional surface models constructed using the 3D analysis software AMIRA v. 5.6.0 (FEI, Visualization Science Group).

**Figure 4.**
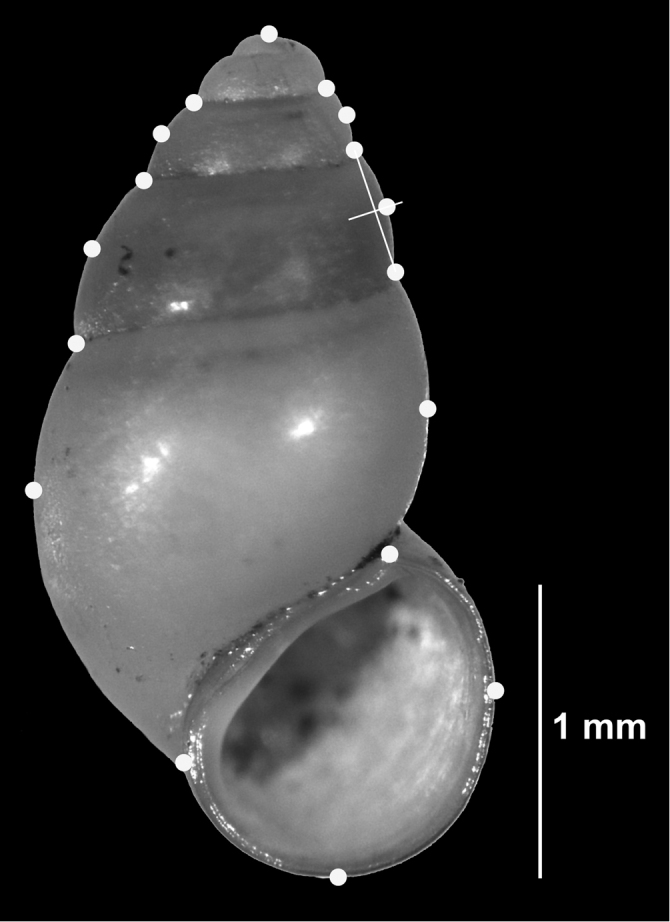
Seventeen landmarks placed on a shell of *Hemistomia fabrorum* from population 39.

For geometric morphometric investigations (Zelditch et al. 2012), shells were photographed at 30-times magnification using a Nikon SMZ 800 stereoscopic microscope (Nikon Corporation, Tokyo, Japan) equipped with a Nikon DS-2M camera and NIS-Elements AR v. 3.2 software (Nikon, Tokyo, Japan). Snails were placed onto a silicone surface for easier positioning, all orientated with the longitudinal axis (columella) parallel to the y-axis. Images were converted into tps format using TPSUTIL v. 1.58 (Rohlf 2013a). 17 landmarks (Fig. 4) were digitized for each individual with TPSDIG v. 2.17 (Rohlf 2013b). In order to check the repeatability of the procedure, it was repeated for ten specimens on two consecutive days. The comparison with Goodall’s F-test in TWOGROUP v. 8 (a program of the IMP suite written by David Sheets: http://www.canisius.edu/~sheets/morphsoft.html) was not significant (p = 0.59) indicating that positioning the shells and placing the landmarks was highly repeatable (cf. Haase and Misof 2009).

In order to visualize the morphological variation, a principal component analysis (PCA) was performed using MORPHOJ v. 1.06a (Klingenberg 2011). COORDGEN v. 8 belonging to the IMP suite was used in order to generate input-data for further analyses with other IMP programs. Based on the PCA, we identified individuals with uncertain morphospecies allocation for further analyzes by canonical variates analysis (CVA) and assignment tests conducted with the IMP program CVAGEN v. 8. For this identification, equal frequency confidence ellipses with a probability of 95% were plotted on the PCA graph. Individuals localized in the intersection of both ellipses and its neighborhood were classified as uncertain and treated as “unknown specimens” in assignment tests including jackknife-assignment (Webster and Sheets 2010). The CVA axes determined by the two known groups were used to assign the unknown specimens to one of the known groups. A pair-wise comparison between the two morphospecies including all specimens previously assigned to either morphospecies with a p-value higher than 0.05 was performed with Goodall’s F-test in TWOGROUP v. 8.

### DNA isolation and sequencing

DNA was isolated using Qiagen’s DNeasy Blood & Tissue Kit (QIAGEN GmbH; Hilden, Germany) in compliance with the manufacturer’s protocol except that we eluted only in 20 μl of AE-buffer. A fragment of the mitochondrial cytochrome c oxidase subunit I gene (COI) was amplified using the primers LCO1490 and HCO2198 (Folmer et al. 1994), the latter modified at position 12 (G→A; Zielske et al. 2011). Polymerase chain reactions were performed in 12.5 μl containing 1.1 μl 10x BH4 buffer (BIOLINE GmbH; Luckenwalde, Germany), 4.4 mM MgCl, 0.3 μM of each primer, 0.2 mM dNTP, 0.5 μl BSA (1%), 0.25 U DNA-Polymerase (BIOLINE), 5–50 ng DNA and water. The PCR conditions were 95 °C for 5 min for denaturation and 40 cycles starting at 94 °C for 60 s, followed by annealing for 90 s with an initial touchdown from 55 to 46 °C with a drop of one degree per cycle, an extension step at 72 °C for 60 s, and 10 min final extension at 72 °C. Amplification products were purified using 4 μl PCR-product and 1 μl Exo-SAP [0.04 μl Exonuclease I (20.000 U/ml; New England BioLabs GmbH; Frankfurt/Main, Germany), 0.15 μl 10x Shrimp Alkaline Phosphatase Buffer (Promega; Madison, WI, USA), 0.81 μl ddH_2_O] per sample. This mix was incubated at 37 °C for 15 min followed by 85 °C for another 15 min. Cycle sequencing was conducted using the Big Dye Terminator Ready Reaction Mix v3.1 (Applied Biosystems, (ABI); Carlsbad, CA, USA) and the PCR primers. The cycle sequencing products were purified using Agencourt’s® CleanSEQ® Dye-Terminator Removal (Beckman Coulter; Beverly, MA, USA) before sequencing on an ABI 3130xl Genetic Analyzer.

### Phylogenetic analyses

COI sequences were edited using the software DNA BASER v. 4.16 (DNA Baser Sequence Assembler v. 4.16 2014). After addition of two outgroup sequences of *Hemistomia nyo* and *Hemistomia andreae* (see Haase and Zielske 2015; Zielske and Haase 2015), the alignment was generated with Clustal W implemented in MEGA v. 6.06 (Tamura et al. 2013) and trimmed to a length of 658 bp. The alignment was screened for potential stop-codons with the software DAMBE v. 5.5.1 (Xia 2013) to check for potential editing errors and nuclear pseudogenes. jMODELTEST v. 2.1.4 (Darriba et al. 2012) identified HKY + I + Γ as best-fitting DNA substitution model according to the Bayesian information criterion. Three phylogenetic analyses were conducted, a maximum likelihood analysis (ML), a bio-neighbor-joining analysis (BNJ), and a neighbor-net analysis. ML was performed using Garli 2.01 (Zwickel 2006). Both optimal tree and bootstrap support were inferred from 500 replicates. The BNJ tree including 5000 bootstrap replicates was constructed in PAUP* v. 4.0b10 (Swofford 2002). Bootstrap support was considered significant if > 75. The neighbor-net was computed with the software SPLITSTREE v. 4.13 (Huson and Bryant 2006) based on the K3ST-model. The optimal model (see above) could not be applied because the program issued undefined distance values.

## Results

### Geometric morphometrics

**Figure 5.**
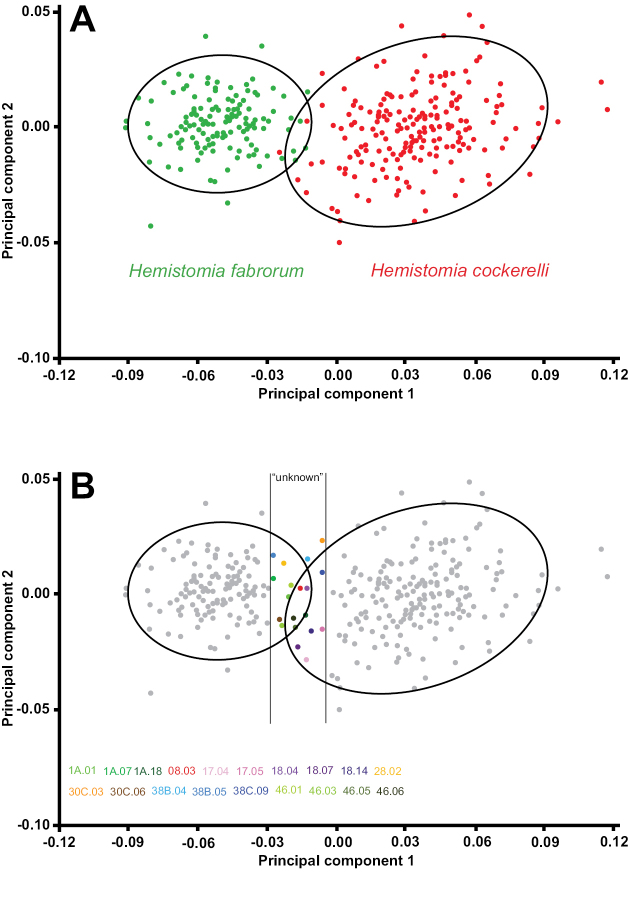
Landmark-based principal component analysis with 95% confidence ellipses. **A** Variation of visually determined *Hemistomia cockerelli* and *Hemistomia fabrorum* **B** Definition of specimens treated as “unknown” in assignment test.

The first two axes of the PCA comparing the morphospecies explained 76.2% of the total morphological variation. The nominal species were fairly well separated along axis 1, however, the 95% confidence ellipses were overlapping. Nineteen specimens within the area of overlap and its neighborhood as defined in Figure 5 were treated as specimens with unknown identity (“unknown”) in further analyses. The CVA defined the set of axes allowing for the greatest possible discrimination of the two groups and axis 1 was highly significant (p < 0.001). These axes were used to assign the unknown specimens. Eight of these specimens were classified as *Hemistomia cockerelli* and 11 as *Hemistomia fabrorum*. All assignments were correct with respect to the initial classification by eye, however, only nine significant. Twelve of the 305 shells with - according to the PCA - unambiguous identity were also assigned correctly but not significantly.

A jackknife test of assignment, a cross-validation procedure, was performed *a posteriori* to assess the robustness of the CV axes and comprised these 305 specimens. In 10,000 replicates, 10% of the specimens (31) were randomly selected as unknown data and assigned to one of the resulting groups of the following CVA analysis based on the remaining 274 shells. 99% of the assignments were correct and significant, 1% correct and not significant, and no shell was incorrectly assigned.

In a subsequent Goodall’s F test both morphospecies were highly significantly (p < 0.001) distinguished. This test included all specimens unambiguously (p > 0.05) assigned to one of the nominal species in the previous CVA-based tests.

### Micro-CT

The μCT-based three dimensional shell surface models revealed, as expected, presence and absence of the palatal denticle in the paratype of *Hemistomia cockerelli* and the topotype of *Hemistomia fabrorum*, respectively (Figs 1, 2). In the specimen from population 9A with shells unusually broad for *Hemistomia cockerelli*, the denticle was well developed, while it was entirely absent in the shell from population 46, a slender *Hemistomia fabrorum*, also confirming the initial classification.

### Phylogenetic analyses

**Figure 6.**
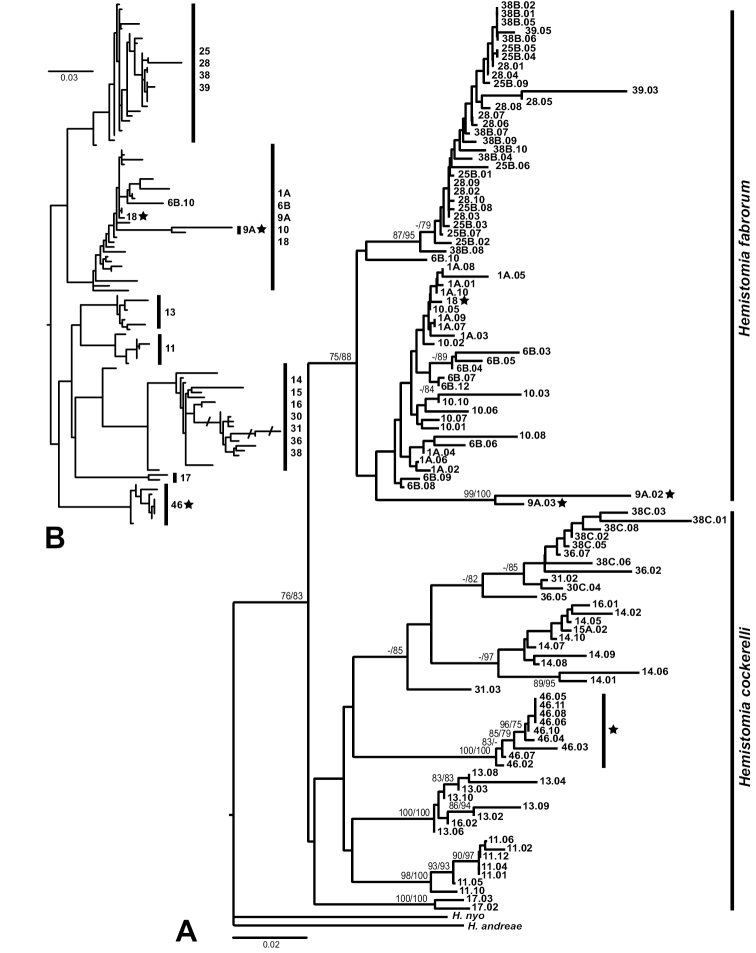
Phylogenetic reconstructions. **A** Bio-neighbor-joining tree based on COI fragment with bootstrap support values from both maximum likelihood and neighbor-joining analyses (ML/BNJ) **B** Maximum likelihood topology. Asterisks indicate “misplaced” individuals. In B only the “misplaced” individuals and one individual (6B.10), whose position differs considerably in both trees, are indicated. Otherwise, only the clade composition is given. Crossed branches are shortened by 50%. The outgroup was pruned from the tree. Scale bars: substitutions per site.

**Figure 7.**
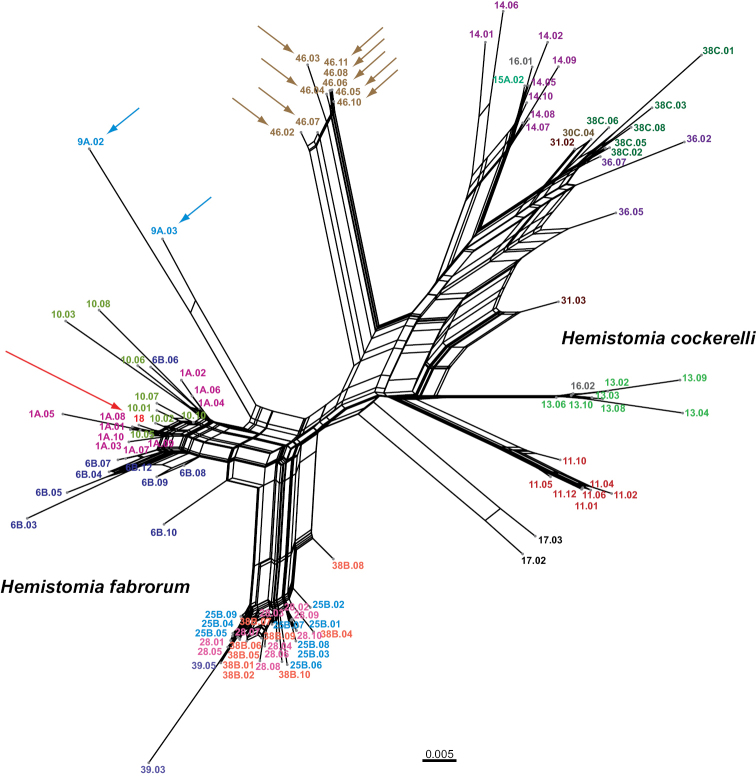
Neighbor-net illustrating conflict in phylogenetic signal of sequence data. Arrows indicate “misplaced” individuals. Scale bar: substitutions per site.

The phylogenetic tree reconstructions inferred very similar relationships. Figure 6 emphasizes the fully resolved BNJ topology, because it had higher bootstrap support than the ML tree except for the clade of population 46. In order to show the general congruence of both reconstructions, the ML topology is given in Figure 6B. The main differences between both optimal topologies were the different positions of the clade of population 46 and individual 6B.10. In the BNJ tree population 46 was nested among specimens allocated to *Hemistomia cockerelli* and 6B.10 sister species of a clade containing sequences from populations 25, 28, 38, and 39. In the ML tree, population 46 was sister group to nominal *Hemistomia cockerelli* and 6B.10 clusterd with other individuals from population 6B. However, when unsupported nodes including those concerning both aforementioned lineages were collapsed, the topologies were practically identical. The ingroup was well supported. It consisted of two main clades of which only the one containing the majority of specimens identified as *Hemistomia fabrorum* received significant bootstrap support. This *Hemistomia fabrorum*-clade included three specimens that were morphologically identified as *Hemistomia cockerelli*, one from population 18 nested with a short branch among individuals from populations 1A and 10, and the two snails from population 9A forming a well supported clade on a long branch. In turn, population 46, morphologically identified as *Hemistomia fabrorum*, clustered with specimens allocated to *Hemistomia cockerelli*. Within both morphospecies, not all populations were monophyletic. The neighbor-net revealed basically the same topology including the shallow position of the specimen from population 18 and the rather basal connections of the snails from populations 9A and 46, respectively (Fig. 7). The network also illustrated the conflicting signal within the two major clades most likely due to lack of data and homoplasy, which explain e.g. the lack of bootstrap support for the *Hemistomia cockerelli*-clade in the tree reconstructions (Fig. 6).

## Discussion

Conflict in phylogenetic/taxonomic signal between different characters is not uncommon among truncatelloidean gastropods, in particular in cases of recent events of speciation and young evolutionary radiations (e.g., Haase 2005; Haase et al. 2007; Zielske and Haase 2014a, b), but also among deeper lineages (Colgan et al. 2006; Wilke et al. 2006; Prié and Bichain 2009). Geometric morphometric analyses and μCT-scans showed that the two nominal species *Hemistomia cockerelli* and *Hemistomia fabrorum* are distinguishable by their shell characteristics. The presorting to morphospecies was 100% in accordance with the final allocation of specimens based on the CVA despite a slight overlap of the variation in the PCA. However, less than 50% of the assignments of specimens with intermediate shape treated as “unknown” specimens were significantly correct. Thus, in face of this remaining ambiguity shell shape alone is not a perfect discriminator. However, both taxa can be unambiguously identified by the presence (*Hemistomia cockerelli*) or absence (*Hemistomia fabrorum*) of the shell denticle (Haase and Bouchet 1998). This denticle is either fully developed or absent. We did not observe intermediate states such as a smaller size. Across *Hemistomia*, this denticle exhibits a considerable variation in shape, size and position and is readily visible through the shell under a dissecting microscope. In several mainly larger species, it is lacking, though (Haase and Bouchet 1998). Also in the genetically “misplaced” specimens from populations 9A, 18 (both morphologically *Hemistomia cockerelli*) and 46 (morphologically *Hemistomia fabrorum*), the denticle was either present or absent.

As the number of individuals misplaced in the phylogenetic analyses was very low and in particular because ncDNA data were lacking, explanations for the incongruences have to remain largely speculative. However, at least for dubious shallow relationships the assumption of a recent event, which can only be introgression through hybridization, is very likely. The specimen identified as *Hemistomia cockerelli* from population 18 was nested among individuals allocated to *Hemistomia fabrorum* from three populations. Since all surrounding, more basal nodes belonged to *Hemistomia fabrorum*, this misplaced specimen most likely has inherited its mitochondria through introgression by hybridization of a female *Hemistomia fabrorum* with a male *Hemistomia cockerelli* in the not too distant past.

In contrast, the misplaced clades consisting of the two specimens from population 9A and the nine individuals of *Hemistomia fabrorum* from population 46, respectively, were connected to deeper, partly unsupported nodes. It is exactly this situation which makes the distinction of incomplete lineage sorting and hybridization difficult (Joly et al. 2009). Assuming hybridization as cause of the topological inconsistencies requires adhoc hypotheses, though. In order to coalesce prior to the completion of speciation a lineage “misplaced” through introgression would have a sole survivor and that only in the species it introgressed into (Joly et al. 2009). Therefore, assuming incomplete lineage sorting as cause for the position of populations 9A and 46 is more parsimonious, although hybridization cannot be ruled out.

In conclusion, despite their partly unresolved relationship *Hemistomia cockerelli* and *Hemistomia fabrorum* may be considered sister species, which are reliably diagnosable by the presence or absence of the palatal denticle, but have not yet fully differentiated in all character complexes investigated. The range of *Hemistomia fabrorum* covers wide parts of southern New Caledonia and largely overlaps with that of *Hemistomia cockerelli*, which is found almost across the entire island (Haase and Bouchet 1998). In contrast to the majority of spring snails in New Caledonia and elsewhere, both species have fairly wide ranges with many island-like populations. This kind of structure has been shown to preserve genetic variation and delay lineage sorting as the effective population size remains large (Slatkin 1991; Thomaz et al. 1996). Most of the investigated *Hemistomia* populations occurred in close geographical proximity. Consequently, interactions between both species cannot be excluded. Interestingly, only in a single locality, 38, were both species found sympatrically. The fifteen individuals sequenced from there fell into the respective correct clades. This suggests that hybridization is probably occurring only rarely. However, three individuals from population 38 were among the 19 with ambiguous shell shape. These comprised also individuals of populations 18 and 46. However, it is impossible to tell whether ambiguities in shell shape had a genetic cause or were due to phenotypic plasticity, which obviously plays an important role in a related species from New Zealand, *Potamopyrgus antipodarum* (Gray, 1843) (Haase 2003; Kistner and Dybdahl 2013; and literature therein).

In order to unambiguously identify the causes of the genetic inconsistencies an even denser sampling design as well as using more genetic markers would be required. The potential role of phenotypic plasticity can only be assessed in common garden experiments. The ambiguous genetic signal also calls for caution for barcoding (Hebert et al. 2003) if this is conducted without morphological control (e.g. Goldstein and DeSalle 2010). Morphologically, *Hemistomia fabrorum* represents the derived conditions regarding the broader shell shape and the lack of a denticle considering that the closest relatives of the pair of species discussed in this account, *Hemistomia andreae* and *Hemistomia nyo*, are hardly distinguishable from *Hemistomia cockerelli* (Haase and Bouchet 1998; Haase and Zielske 2015).
